# TEVAR and EVAR, the unknown knowns of the cardiovascular hemodynamics; and the immediate and long-term consequences of fabric material on major adverse clinical outcome

**DOI:** 10.3389/fsurg.2022.940304

**Published:** 2022-08-30

**Authors:** Sherif Sultan, Yogesh Acharya, Osama Soliman, Juan Carlos Parodi, Niamh Hynes

**Affiliations:** ^1^ Department of Vascular and Endovascular Surgery, Western Vascular Institute, University Hospital Galway, National University of Ireland, Galway, Ireland; ^2^ Galway Clinic, Doughiska, Royal College of Surgeons in Ireland and the National University of Ireland, Galway Affiliated Hospital, Galway, Ireland; ^3^ CÚRAM-CORRIB-Vascular Group, National University of Ireland, Galway, Ireland; ^4^ Department of Vascular Surgery and Biomedical Engineering Department, University of Buenos Aires, Buenos Aires, Argentina

**Keywords:** abdominal aortic aneurysm (AAA), endovascular aneurysm repair (EVAR), thoracic endovascular aneurysm repair (TEVAR), stent-Graft material, cardiovascular outcome

## Abstract

This review discusses the impact of endovascular aneurysm repair on cardiovascular (CV) hemodynamics and the role of stent-graft material, i.e., polytetrafluoroethylene (PTFE) vs. polyester in post-procedural outcomes. Endovascular aneurysm repair has been widely employed in the last decades for thoracic and abdominal aneurysm repair. However, aortic endografts are stiff and alter the native flow hemodynamics. This failure to simulate the native aorta could lead to added strain on the heart, manifesting as increased left ventricular strain, higher pulse pressure, and congestive heart failure later. This could result in adverse CV outcomes. Also, evidence is mounting to support the implication of stent-graft materials, i.e., PTFE vs. polyester, in adverse post-procedural outcomes. However, there is an absence of level one evidence. Therefore, the only way forward is to plan and perform a randomised controlled trial to demonstrate the alterations in the CV hemodynamics in the short and long run and compare the available stent-graft materials regarding procedural and clinical outcomes. We believe the best solution, for now, would be to reduce the stented length of the aorta. At the same time, in the longer term, encourage continuous improvement in stent-graft materials and design.

## Introduction

Compared to open surgical repair, endovascular repair of the thoracic and abdominal aorta has been shown to reduce early perioperative morbidity and mortality (1, 2). However, this advantage is not maintained later due to an increment in cardiovascular (CV) complications secondary to arterial stiffening by endograft (3). It is, therefore, essential to be aware of the impact of endograft design, their relative configuration, and stiffness compared to the native aorta (3–5). Also, the role of the endograft composition and structural design (i.e., endograft materials—polyester vs. polytetrafluoroethylene (PTFE), stent wires—nitinol vs. stainless-steel stent vs. cobalt-chromium) on the post-procedural outcomes needs to be acknowledged (3–9).

This review discusses the impact of endograft on CV hemodynamics in the first half and, subsequently, in the second half, the impact of stent-graft material, i.e., PTFE vs. polyester, in post-procedural outcomes, including post-implantation syndrome.

## Materials and methods

This study was conducted through a non-structured online literature search (PubMed, Google Scholar and EMBASE) using the keywords—“Cardiovascular Hemodynamics,” “Cardiovascular Complications,” “Cardiovascular Outcomes,” “Abdominal Aortic Aneurysm,” “AAA,” “Endovascular Repair,” “TEVAR,” “Thoracic Endovascular Aneurysm Repair,” “EVAR,” “Endovascular Aneurysm Repair,” “Endograft,” “Stent-graft material,” “PTFE,” “Polytetrafluoroethylene,” “Polyester,” and “Outcome.” No selective restrictions were made on the type of studies, publication year and language. A secondary reference search was used to obtain further studies.

## Impact of EVAR and TEVAR on cardiovascular haemodynamics

Aortic endografts are stiffer than the native aorta, and even the best available contemporary endograft design could potentially alter the flow haemodynamics (3–5). Studies have shown that aortic endografts could significantly reduce coronary perfusion by elevating systolic blood and pulse pressure (5–7, 10). These patients suffer on and off chest pain and systolic hypertension from early postoperative days. However, the broader CV community lack insight regarding cardiac remodelling post-aortic stents as interventionalists primarily focus on endo-graft adaptation rather than hemodynamic alterations. Furthermore, our follow-up protocols are based only on close supervision for endograft migration, detecting endoleak and aortic sac regression, for which we are not afraid of further stenting and coiling, thereby creating a stiffer aortic wall, which could further compromise cerebral, cardiac, renal, and mesenteric perfusion (3–7).

## Pathophysiology

**Figure 1:**
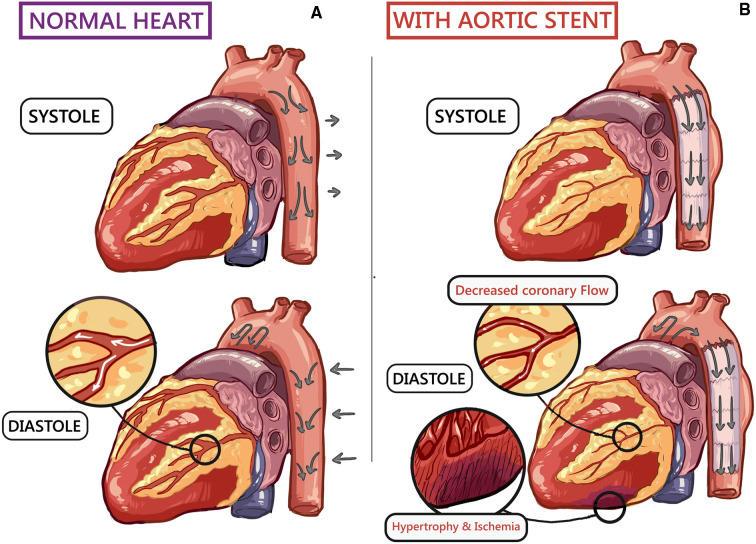
A schematic human heart diagram showing (10): (**A**) normal cardiovascular hemodynamics during systole and diastole. (**B**) Endograft in the thoracic aorta increases arterial stiffness, causing left ventricular (LV) strain and subsequent hypertrophy. Furthermore, impaired LV filling decreases coronary blood flow, resulting in non-occlusive ischaemia.

The aorta receives the left ventricle (LV) stroke volume in systole, which is distributed peripherally through the stored aortic elastic forces gained during diastole. This aortic compliance and blood flow through the aorta is best represented by the “Windkessel effect” (Figure 1) (10).

Windkessel effect impact both the heart and the peripheral circulation. Aortic compliance decreases the LV afterload. Furthermore, blood collected within the distended aorta helps to enhance coronary perfusion.

A mismatch between the native aortic to endograft compliance could manifest as adverse CV outcomes. Any change in the Windkessel effect could significantly increase the LV burden, resulting in adaptative LV hypertrophy and loss of ventricular-arterial coupling (11, 12).

As aortic endografts are less compliant than the native aorta, insufficient compliance results in a surge in hemodynamic shifts that impair CV homeostasis (3, 5, 8). Arterial stiffening results in elevated systolic blood pressure but lowers diastolic blood pressure, further exacerbating LV afterload, resulting in mal-perfusion of the coronaries. These changes contribute to LV hypertrophy, coronary ischemia, and arterial wall tissue fatigue, which are independent risk factors for CV morbidity and mortality (10–14).

Rong et al. (15) demonstrated amplification of circumferential strain in the descending thoracic aorta, paralleling distensibility by using intra-operative transoesophageal echocardiography to study the effect of endograft on the haemodynamic alteration. They showed that prosthetic replacement of the ascending aorta could interfere with the propagation of energy to the distal aorta resulting in adverse aortic remodelling. These results explain the development of resistant systolic hypertension post-TEVAR/EVAR with shortness of breath (SOB) and intermittent chest pain.

The impact of aortic flow dynamics on the LV function has been studied in experimental models (16), animals (17) and clinical studies (18). These studies support the Windkessel theory to establish the role of aortic capacitance in resultant ventricular size and function. However, the stented aorta loses its elasticity following simple and/or complex endovascular procedures, like TEVAR, FEVAR, BEVAR, and ChEVAR, failing the Windkessel effect. This failure of the Windkessel effect and change in pulse wave propagation multiplies a substantial workload for the LV putting an extra strain on the aortic valve's functioning. The resultant adaptative LV hypertrophy will manifest as CV complications (10–12).

**Figure 2:**
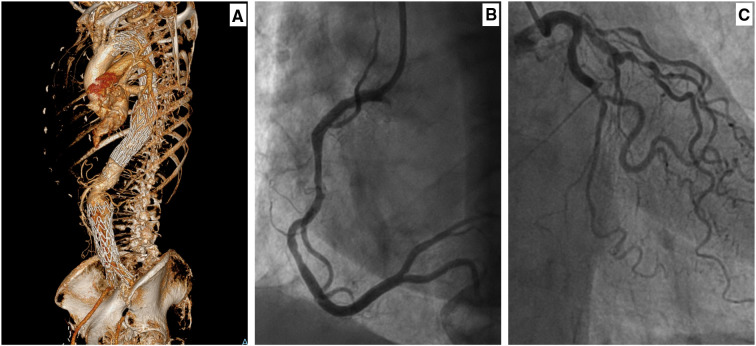
A female in her seventies with a saccular aneurysm in the descending thoracic aorta (10). She had thoracic endovascular aortic repair (TEVAR) in 2015 and subsequently underwent endovascular repair of her infrarenal aortic aneurysm in 2018. Her background history included ex-smoker, hypertension, lipid disorder, and right femoral-popliteal percutaneous transluminal angioplasty. (**A**) A 3D-CTA reconstruction, showing thoracic endograft. Following the TEVAR, the patient complained of intermittent chest pain and shortness of breath. A coronary angiogram was performed after her symptoms worsened. (**B**) Coronary angiogram (right main coronary artery) with no evidence of occlusive coronary disease. (**C**) Coronary angiogram (left main coronary artery) with no evidence of occlusive coronary disease.

The negative impedance due to endograft and LV strain will cause a decrease in diastolic systemic BP and reduces coronary blood flow and myocardial ischemia without coronary artery stenosis (10, 13). Sultan et al. (3, 5, 8, 10, 14–18) documented cardiac dysfunction on the postoperative echocardiograms of TEVAR/EVAR cases, with moderate LV hypertrophy and diastolic dysfunction. This is manifested by an increase in proBNP, which supports myocyte stretching and ventricular strain. Moreover, there was a significant troponin rise without coronary artery stenosis. Furthermore, the coronary angiography confirmed the absence of the coronary blockage, which supports the alternative explanation of coronary hypoperfusion following reduced diastolic pressure (Figure 2) (10).

Aortic compliance mismatch and hemodynamic alterations will be more evident after increasing the length of the stented aorta, for example, following combined TEVAR and EVAR. As such, endograft tend to adapt to these increments in shear stress. Studies have shown gradual endograft dilation after open surgical repair (3.2% per year post repair) (19–22). This could sometime result in excessive strain on the fabric architecture and the development of new aneurysms (10, 13).

Aortic integrity affects CV outcomes. This is evident in acute aortic syndrome, where CV complications are the main culprit for the late rehospitalisation after discharge (23, 24). Weiss et al. (24) showed nonfatal CV events and heart failure in patients with aortic dissection, intramural hematoma and penetrating aortic ulcer. These outcomes strengthen the need for long-term CV follow-up following endovascular aortic repair.

## Pulse wave velocity

One of the ways to measure the impact of endograft stiffness on aortic impedance is to measure PWV.

PWV represents arterial stiffness, as higher arterial stiffness is seen with higher PWV. Subsequent increment in PWV increases the CV morbidity and mortality. Interesting, PWV could increase within a few hours of TEVAR and/or EVAR (21, 22, 25). TEVAR and EVAR increase the PWV by 2–5 and 1–3 m/s; however, a combined TEVAR/EVAR will result in an increment of 3–8 m/s (21, 22, 25). Blacher et al. (21) acknowledged that 1 m/s of PWV increment would double the all-cause mortality.

TEVAR has been shown to increase LV stroke work by 26% (26). Van-Bakel et al. (16) showed that structural stiffness increased from 10.2 to 154.6 MPa/mm post-TEVAR. This is a 15-fold increase in workload for the heart within minutes of deployment of a TEVAR; as the heart was not preconditioned, the CV haemodynamic changes accelerate over time. Furthermore, increment in vascular stiffness with endograft results in ventricular diastolic dysfunction, thereby negatively impairing exercise tolerance amongst patients with lower LV distensibility (11, 12, 27).

We contemplated that PWV could be utilised in risk assessment in the peri-operative period post-TEVAR/EVAR. Risk stratification provides an opportunity to address hemodynamic alterations and modulate the CV risk (21, 22, 25).

## Impact of stent-graft materials on post-procedural outcomes and post-implantation syndrome

A 4-dimensional strategy (14) is necessary to manage complex aortic pathologies as altered haemodynamic forces increase wall shear stress and impair blood flow, causing flow turbulence, pressure gradients, and blood viscosity increment. It involves the morphological adjustment and hemodynamic milieu of natural body forces since the resultant flow disturbance affects the management outcome (14).

Sultan et al. (10) documented that patients with combined TEVAR and EVAR can develop adaptive LV hypertrophy and diastolic dysfunction. This could result in a clinical picture like lower limb oedema, SOB, and chest pain with a normal coronary angiogram (Figure 2) (10).

The increase in aortic stiffness post-TEVAR could be seen earlier than EVAR due to proximity to the heart (28). However, the length of the stented aorta also matters. Combined TEVAR and EVAR in this regard have earlier and more pronounced impacts (10). Nonetheless, it is prudent that all the available endograft are less compliant than the native aorta (16, 29).

In terms of the endograft material, the Liapis group (30) showed that endograft made with polyester results in a threefold increment in PWV than PTFE.

We witnessed that TEVAR patients developed the abdominal aortic disease after endograft implantation (10). In these patients, worsening hypertension and late CV complications were potentiated by having a stiff tube in the aorta. This necessitates studies that specifically focus on CV complications post-aortic endograft. Also, it is possible that careful analysis of the endograft based registry could answer that question at present.

Patients with connective tissue disorder, like Marfan's syndrome, have a defect in the aortic wall, which could further complicate the compliance mismatch and result in aneurysmal dilatation.

Suppose this is explained to young trauma patients post aortic transection who underwent emergency TEVAR. This will result in unexplained congestive cardiac failure and dilated cardiomyopathy post-TEVAR in many young patients following aortic trauma.

Modified and complex endovascular techniques (BEVAR, FEVAR, PETTICOAT (31), STABILISE (32), FLIRT (33), Candy Plug (34), Knickerbocker (35), and Kinetic Elephant trunk (36) could provoke additional aortic wall stress. The risk of these CV complications increases more when stents are deployed closer to the heart and aortic valve (7, 36–39).

We acknowledged in our previous publications that the best solution is to reduce the length of the stented aorta through a “Staged hybrid single lumen reconstruction (TIGER)” protocol (3, 4, 26, 40, 41). TIGER protocol combines open abdominal aortic repair with thoracic aortic stenting. For this, we first create a single lumen from supra celiac, infra-diaphragmatic aorta to bilateral common iliac arteries through visceral arteries open surgical patching and subsequently perform TEVAR after that (4). With the reduction in the stented length of the aorta, the TIGER technique has shown that fewer aortic stents and grafts have superior long-term CV outcomes (3, 4, 14, 41).

Cardiac dysfunction following TEVAR/EVAR is a complex challenging scenario for CV interventionalist (10, 29). Therefore, it is essential to contemplate the compliance mismatch and long-term adverse CV outcomes. The nearer to the heart the endograft is deployed, the worse is the effect. The way to the future is to respect the aorta as an active organ, not a mere conduit.

The ideal design of the aortic endograft should resemble the native aorta in terms of its flexibility and hemodynamic impedance. The stent-graft polymers should be lightweight but strong and resilient and capable of withstanding the impact of normal pulsatile high flow arterial blood pressure. However, ePTFE and polyester are synthetic polymers that are relatively stiff and rigid compared to the native aorta (42–47).

There are no RCTs or CCTs to validate post-procedural outcomes following EVAR/TEVAR with specific stent-graft materials. Although not powered to demonstrate the difference in outcomes based on endografts, the EVAR I trial showed reduced major adverse clinical events (MACEs) with the PTFE based GORE Excluder graft (48, 49). Furthermore, direct comparisons are further complicated by the heterogeneity of individual manufacturers' differences in endograft design and procedural deployment techniques (50–53).

Consequently, it is difficult to accurately predict the impact of the stent-graft materials on hemodynamic alteration. PWV is a surrogate marker that demonstrates changes in stiffness following EVAR. Liapis et al. (30) showed that post-EVAR with polyester endografts, there could be a threefold increase in PWV compared to PTFE. PTFE endografts have been reported to offer significantly stronger resistance to dilatation than polyester-based endografts initially, albeit this advantage is lost over time (54). Similarly, there were lower complications with PTFE grafts (55). However, there are no reports of apparent long-term advantages.

PTFE-based endografts, compared to polyester, are associated with a lower incidence of post-implantation syndrome (PIS). PIS has been reported in up to two-thirds of the patients following TEVAR/EVAR (56), resulting in acute liver and/or multiple-organ failure (57–62). Ito et al. (56), Voûte et al. (63), and Sartipy et al. (64) implicated polyester-based endografts in the development of postoperative pyrexia, PIS, and more extended hospital stay post-EVAR compared to the PTFE-based endografts.

Ferreira et al. (65) suggested a probable interlink between PIS and increased CV mortality as polyester-based endografts increased inflammatory responses that caused endothelial damage. Higher serum IL-8 levels support this as IL-8 has pro-inflammatory and pro-tumoural functions. Also, IL-8 implicates the potential of polyester-based endografts; however, it is yet to be established (66–68).

Similarly, the use of polymers in EVAR within PTFE fabric has been controversial, and the polymer-based endografts, like Nellix (Endologix Inc., Irvine, CA, USA) and Ovation iX (Endologix Inc., Irvine, CA, USA) abdominal stent graft system device, were subsequently removed from the market (69, 70). They failed in short and mid-term follow-ups because of an inadequate proximal fixation with continuous pressure necrosis on the aortic sac for Nellix and aortic neck wall for the Ovation (69, 70). Any technology that uses embedded high inflation rings (Ovation iX) or balloons/endobags (Nellix) must be contraindicated, as the aorta is an organ that must be respected. Any attempt to manage it as a mere conduit is destined to fail.

The Alto device is a newer generation of the Ovation Xi platform, which combines PTFE limbs with the main body with polymer-filled rings to assist with sealing the proximal aortic neck (69). The technology is evolving, and there is limited long-term data on performance.

There have been studies looking at the effect of Ovation on PWV, which found no increment, but they did not compare it to other devices (71). However, PIS with polymer-based EVAR has the equivalent outcome as PTFE-based endografts with the added complications of aggravated PIS due to activation of TNF and monocytes at the site of high inflation balloons and/or rings (63–65).

## The future

We must innovate in creating intelligent, compliant, durable endoprostheses that do not require any maintenance or follow up. It will be manufactured by a “Bio-inspired Smart Self-Healing Material with Autonomous and Non-Autonomous Nanoparticles” as a nano-carrier for self-healing, self-repairing and self-assembly systems. These elements are vital components for durable smart endoprostheses.

The intelligent endoprosthesis will adapt itself to prevent tissue ingrowth into its' microstructure, preventing rigidity and maintaining distensibility. Therefore, the Smart endoprosthesis will retain the ability to expand in systole and collapse in diastole. After implantation, it gives back the elastic recoil to the heart, creating an almost standard aortic flow curve.

Bio-active-bio-inspired scaffolds will allow the smart endoprosthesis to be more robust and fault-tolerant. Transverse and longitudinal crimping that expands in systole and contracts in diastole will mimic the elastic recoil of the aorta. Hence it will abolish CV hemodynamic consequences of adaptive LV hypertrophy, the wide pulse pressure, the congestive heart failure and the renal impairment.

This paradigm shift towards utilising bio-inspired smart self-healing materials to build smart endoprosthesis capable of advanced self-healing during the functional lifetime of the endograft is a disruptive technology and will augment bio-convergence (72).

Intelligent bio-inspired endoprosthesis will lengthen product lifetime and abolish the need for follow-up or re-interventions. It is an intelligent green environmental friendly endoprosthesis that requires no service—a “TESLA like scenario”.

## Conclusion

There is increasing evidence of adverse hemodynamic alteration post-TEVAR/EVAR. Furthermore, evidence to support the implication of specific stent-graft materials, i.e., PTFE vs. polyester, in adverse post-procedural outcomes following endovascular repair of AAA is mounting. Interventionalists must respect the aorta as an active organ, not a mere conduit. The best solution in the short term could be to reduce the stented length of the aorta while in the longer-term encouraging continuous improvement in stent-graft materials and design. In the absence of level one evidence, the only way forward is to plan and perform an RCT or CCT to compare the available stent-graft materials regarding procedural and clinical outcomes.
